# Stability enhancement of lycopene in *Citrullus lanatus* extract via nanostructured lipid carriers

**DOI:** 10.1002/fsn3.2156

**Published:** 2021-01-31

**Authors:** Jutiporn Sirikhet, Wisinee Chanmahasathien, Araya Raiwa, Kanokwan Kiattisin

**Affiliations:** ^1^ Department of Pharmaceutical Sciences Faculty of Pharmacy Chiang Mai University Chiang Mai Thailand; ^2^ Nutraceuticals, and Cosmeceuticals Faculty of Pharmacy Innovation Center for Holistic Heath Chiang Mai University Chiang Mai Thailand

**Keywords:** *Citrullus lanatus*, lycopene, nanostructured lipid carriers, stability, watermelon

## Abstract

Lycopene is one of naturally occurring carotenoids in plants including watermelon *(Citrullus lanatus)*. Heat, light, and oxygen effect on lycopene isomerization and degradation. Nanostructured lipid carriers (NLCs) are drug delivery system which can enhance the stability of active compound. Therefore, this study aimed to develop watermelon extract loaded in NLCs for lycopene stability improvement. The NLCs were prepared using a hot homogenization technique. Cocoa butter was used as solid lipid. Grape seed oil was used as liquid lipid. Span^®^ 80 and Plantasens^®^ HE20 were used as an emulsifier. The selected unloaded NLCs contained solid lipid to liquid lipid at the ratio of 3:1 and 10% (w/w) of total lipid. The particle size of watermelon extract‐loaded NLCs (WH‐loaded NLCs) was 130.17 ± 0.72 nm with low PDI and high zeta potential. It also presented high entrapment efficiency. For stability study, the WH‐NLC3 could enhance stability and maintain lycopene content after stability test. It exhibited the highest values of lycopene content (83.26 ± 2.30%) when stored at 4°C. It also possessed a prolonged release pattern over 48 hr. Therefore, the NLCs could improve stability and release profile of lycopene from watermelon extract.

## Graphical abstract

The WH‐loaded NLCs were successfully prepared to enhance the stability and prolonged release profile of lycopene. It is a promising delivery system with a safe ingredient and an easy process for improving the stability of WH extract and other unstable extracts. Furthermore, it can further study for use in industry or high scale production.

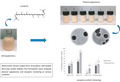

## 1 INTRODUCTION

Lycopene is found in red fruits and vegetables especially tomato, and pink grapefruit including watermelon (Celli et al., 2016). Recently, research showed that watermelon contained many carotenoids, such as lycopene, beta carotene, and lutein (Hena et al., 2016). Nowadays, natural active ingredients have been used in pharmaceutical, nutraceutical, cosmeceutical, and cosmetic products due to health benefits, their biocompatibility and safety. There are varieties of fruits in Thailand which are tropical plants and rich in antioxidant compounds such as polyphenols, vitamins, and carotenoids (Rivera‐Pastrana et al., 2010). The lycopene content in watermelon was higher than other fruits and vegetables, and it ranges from 2.30 to 7.20 mg/100 g fresh weight (Perkins‐Veazie & Collins, 2004). Another study showed that average lycopene content in watermelon was 47–68 µg/g fresh weight, which showed 60% higher than fresh tomatoes (30 µg/g fresh weight) (Gomes et al., 2013). In addition, it can harvest in all seasons and generally found in market and cheap price.

Lycopene belongs to the carotenoids that also important free radical scavenger that protects the organism from overexposure to damaging UV light. Naturally antioxidant and UV‐blocking capabilities of lycopene make it a valuable active compound against skin aging (Rao & Rao, 2007). Lycopene could improve skin protection against UV radiation better than beta carotene. Previous research showed that the use of high‐lycopene foods in diet could reduce the skin damage caused by UV‐A and UV‐B exposure (Stahl & Sies, 2012). Moreover, lycopene could reduce erythema or skin redness (Tapiero et al., 2004). Lycopene is found to concentrate in the skin, adrenal, prostate, and testes where it protects against cancer and could reduce LDL cholesterol levels. However, lycopene is unstable molecule. All‐*trans*‐isomer of lycopene is sensitive to isomerization and also oxidation into *cis*‐isomer (Regier et al., 2005). Health benefits and bioactivity are also reduced by isomerization and oxidation. Because of many conjugated double bonds in its molecule, it is very susceptible to oxidation when exposed to air, light, and heat during storage (Sharma & Le Maguer, 1996; Zechmeister et al., 1943). In addition, it is water‐insoluble compound and hardly permeates into the skin. Therefore, nanocarriers are used to improve lycopene stability and skin penetration (Ascenso et al., 2011). The structure of lipid carriers could entrap hydrophilic, hydrophobic, or macromolecular compounds (Ainbinder et al., 2010). The nanostructured lipid carriers (NLCs), which could be achieved by mixing solid lipid with partial liquid lipid, have become an interesting delivery system for cosmetic and pharmaceutical products. It is an alternative delivery system to improve the stability, solubility as well as bioavailability and protect sensitive bioactive from unpleasant conditions. Moreover, it could enhance a high loading capacity, control the release rate of bioactive compound and its targeting, especially for lipophilic compounds. Many water‐insoluble active compounds were reported to be successfully incorporated in NLCs and showed effective skin permeation (Hentschel et al., 2008; Pardeike & Müller, 2007). The NLCs had many advantages over other traditional carrier systems such as occlusive effect, improve the absorption of active compound and prepare large‐scale production. Moreover, it can be used for photoprotective agent. Hence, the NLC is the suitable carrier for lycopene to enhance the absorption and stability (Riangjanapatee & Okonogi, 2012). Therefore, the aims of this research were to develop watermelon extract‐loaded NLCs to enhance stability and releases profile of lycopene.

## 2 MATERIALS AND METHODS

### 2.1 Plant material preparation

Seeds and peel of watermelon fruits (Kinnaree) were removed and blended with blender to get condensed watermelon juice. Then, it was frozen at −40°C. The frozen sample was placed in a freeze dryer and then vacuum‐dried for 24 hr. After drying, sample was collected in amber bottle at 4°C.

### 2.2 Chemical materials

Lycopene, 2,2‐diphenyl‐1‐picrylhydrazyl radical (DPPH), 2,2'‐Azobis(2‐amidinopropane) dihydrochloride (AAPH), and ferric chloride (FeCl_3_.6H_2_O) were purchased from Sigma‐Aldrich Inc., USA. Cocoa butter was purchased from Tropicalife CO., Ltd., Thailand. Methanol (HPLC grade), tetrahydrofuran (HPLC grade), deionized water (HPLC grade) 95% ethanol (Analytical grade), acetone (Analytical grade), hexane (Analytical grade), dimethyl sulfoxide (Analytical grade), and hydrochloric acid (Analytical grade) were purchased from Labscan Ltd., Ireland. Ammonium thiocyanate (Analytical grade) was purchased from Kemaus, Australia. Linoleic acid was purchased from Fluka Buchs, Switzerland. Plantasens^®^ HE20 (Cetearyl glucoside, Sorbitan olivate) was purchased from Clariant CO., Ltd., Thailand. Grape seed oil, almond oil, Avocado oil, olive oil, sesame oil, sorbitan oleate (Span^®^ 80), polysorbate 20 (Tween^®^ 20), and propylene glycol were purchased from Namsiang CO., Ltd., Thailand.

### 2.3 Extraction

The freeze‐dried watermelon was extracted by Soxhlet extraction with hexane: acetone: ethanol (2:1:1) (Haroon, 2014). The details of extraction are as follows: The freeze‐dried watermelon powder (100 g) was placed in cellulose extraction thimbles and covered with cotton wool. The thimble was placed in the Soxhlet extraction unit and extracted for 12 hr. Extraction was done in the dark to minimize lycopene degradation. Solvent was evaporated by vacuum rotary evaporator. The concentrated watermelon extract (WH) was weighed and calculated percentage of yield.

(1) $$\%\text{Remaining}=\frac{\text{The}\;\;\text{amount}\;\;\text{of}\;\;\text{lycopene}\;\;\text{in}\;\;\text{formulation}\;\;\text{after}\;\;\text{stability}\;\;\text{test}\times \text{1}00}{\text{The}\;\;\text{amount}\;\;\text{of}\;\;\text{lycopene}\;\;\text{in}\;\;\text{formulation}\;\;\text{at}\;\;\text{initial}}\%yield=\left(\text{weight of extract}/\text{dry weight of plant}\right)\times \text{1}00)$$

### 2.4 Analysis of lycopene content by high‐performance liquid chromatography (HPLC)

WH extract and lycopene standard was dissolved in methanol: tetrahydrofuran (1:1) and filtered through nylon syringe filter (0.22 µm, 13 mm) until it turned to clear solution. The nonpolar C‐18 analytical chromatographic column (COSMOSIL 5 C_18_, MSII, 4.6 ID × 250 mm) was used as stationary phase for analyzing lycopene content in watermelon extract. The mobile phase consisted of methanol: tetrahydrofuran: water at the ratio of 60:33: 7 (%v/v). The analysis was performed with isocratic mode. The injection volume (10 µl) was eluted with mobile phase at flow rate 1.5 ml/min. The lycopene chromatogram was detected at 475 nm by high‐performance liquid chromatography (HPLC, Shimadzu, Japan). The HPLC chromatograms were quantitatively analyzed based on peak area measurement (Okonogi & Riangjanapatee, 2015).

Preparation of standard calibration curve, a stock solution of commercially pure lycopene (Sigma‐Aldrich) was prepared by dissolving 1 mg of lycopene in 5 ml of methanol: tetrahydrofuran (1:1) and then stock solution was diluted with methanol: tetrahydrofuran (1:1) to prepare concentration of 0 (blank), 0.03125, 0.0625, 0.125, 0.25, 0.5, 1, 2.5, 5, 7.5, 10, and 20 µg/ml. Aliquots of the standards were analyzed by HPLC in triplicates.

### 2.5 Stability of lycopene in watermelon extract

The stability of watermelon (WH) extract was tested by keeping in microcentrifuge tube and stored under various conditions including heating–cooling cycling (HC) at 4 ± 0.5°C for 48 hr and moved to hot air oven at 45 ± 0.5°C for 48 hr (one cycle) for 6 cycles, room temperature (RT or 30 ± 0.5°C), 4 ± 0.5°C and 45 ± 0.5°C for 3 months. After HC and 3 months of storage, each WH extract was dissolved in methanol : tetrahydrofuran (1:1) and filtered through nylon syringe filter (0.22 µm, 13 mm) until it turned to clear solution. The lycopene content of WH extract was analyzed by HPLC (HPLC, Shimadzu, Japan) before and after the stability test.

### 2.6 Preparation of NLC formulation

Unloaded NLCs composed of mixture of solid and liquid lipids. Cocoa butter was used as solid lipid. Liquid lipid was chosen from sesame oil, olive oil, almond oil, avocado oil, and grape seed oil. It was selected by solubility and antioxidant activity. Span^®^ 80 and Plantasens^®^ HE20 were used as emulsifying agent in formulation.

#### 2.6.1 Solubility study of watermelon extract in liquid lipid

Watermelon extract (0.5 g) was dissolved in each liquid lipid which it was added until extract completely dissolved. The amount of liquid lipid required to solubilize the WH extract was determined.

#### 2.6.2 Determination of antioxidant activity of liquid lipid

##### DPPH radical scavenging capacity assay

This method is based on reduction of the DPPH radical (Lin & Chang, 2000). A 20 µl of each oil was added to 180 µl of DPPH solution. After mixing, it was incubated at room temperature for 30 min. Then, the absorbance was measured at 520 nm using microplate reader (SPECTROstar Nano 220–1000 nm, BMG LABTECH, USA). Free radical scavenging activity was calculated as % inhibition from this equation:

(2) $$\text{Percentage}\;\;\text{of}\;\;\text{inhibition}\left(\%\right)=\left(A_{\text{control}}‐A_{\text{test}}\right)/A_{\text{control}}\times \text{1}00$$

where A_control_ is the absorbance of the control reaction at 520 nm and A_test_ is the absorbance of the test reaction at 520 nm.

##### Lipid peroxidation inhibition assay

Each oil was taken in a test tube and added with 1.4 ml of linoleic acid in methanol, 1.4 ml of phosphate buffer (pH 7), and 0.7 ml of distilled water and then incubated in water bath at 45°C in the dark condition for 4 hr. After 4 hr of incubation, 50 µl of this solution was added in test tube with 5 ml of 7% methanol in deionized water and 50 µl of 10% ammonium thiocyanate. Lastly, 50 μl of 20 mM ferrous chloride in 3.5% hydrochloric acid was added to the mixture for 3 min. The peroxide levels were determined by reading the absorbance at 500 nm using UV–visible spectrophotometer (UV‐2450, Shimadzu, Japan) (Rivero‐Perez et al., 2007). The percentage of inhibition was calculated using this formula:

(3) $$\text{Percentage}\;\;\text{of}\;\;\text{inhibition}\left(\%\right)=\left(A_{\text{control}}‐A_{\text{test}}\right)/A_{\text{control}}\times \text{1}00$$

where A_control_ is the absorbance of the control reaction at 500 nm and A_test_ is the absorbance of a test reaction at 500 nm.

#### 2.6.3 Development of unloaded NLCs

NLCs were prepared by high pressure homogenization based on the previous method with some modifications (Riangjanapatee & Okonogi, 2012). The NLCs were prepared with appropriate total lipid (10%) and solid lipid to liquid lipid ratio (3:1) following previous study (Sirikhet et al., 2019). Cocoa butter (7.5%w/w) was used as solid lipid, and grape seed oil (2.5%w/w) was used as liquid lipid. Later, the effect of emulsifiers in formulations was studied. Span^®^ 80 and Plantasens^®^ HE20 in ratio 1:1 was varied concentration with 2.5, 5 and 7% (w/w) (Table 1). In brief, lipid phase (liquid lipid and solid lipid) and water phase were separately preheated in water bath until 75°C and 80°C, respectively. To obtain pre‐emulsion, the water phase was added into lipid phase and stirred continuously with stirring speed of 3,000 rpm. The pre‐emulsion was reduced particle size by high pressure homogenizer (DRAWELL, Model: JG‐1A) with appropriate pressure (500 bars) and 8 cycles to obtain NLC formulation.

**TABLE 1:** Amount and type of emulsifiers used in unloaded NLCs

Formulation	Mixed surfactants (%w/w)	Span^®^ 80 (%w/w)	Plantasens® HE20 (%w/w)
A1	2.5	1.25	1.25
A2	5.0	2.50	2.50
A3	7.0	3.50	3.50

#### 2.6.4 Characterization of unloaded NLCs

The particle size, polydispersity index (PDI), and zeta potential of unloaded NLCs were measured by Zetasizer (Zetasizer^®^ ZS, Malvern Instruments Ltd., UK). Each formulation was diluted at a ratio of 1:100 with deionized water and then the diluted sample was measured the particle size and polydispersity index (PDI) at room temperature (25 ± 0.5°C) with the detection angle of 173° (Nitthikan et al., 2018). The measurement was performed in triplicate. The formulation with good appearance, small particle size, and narrow PDI value was selected for loading the WH extract.

### 2.7 Development of watermelon extract‐loaded NLCs

Watermelon extract‐loaded NLCs (WH‐loaded NLCs) were prepared by adding the WH (1% w/w) into selected unloaded NLCs. The selected unloaded NLCs consisted of solid lipid and liquid lipid with the ratio of 3:1 and 7% emulsifier as shown in Table 2. The pre‐emulsion occurred after mixing the oil phase and water phase and then generated to NLCs using the high pressure homogenizer with 500 bars and 8 cycles. The WH‐loaded NLCs were cooled down to room temperature.

**TABLE 2:** Compositions of watermelon extract‐loaded NLCs

Composition	% w/w
Cocoa butter	7.5
Grape seed oil	2.5
Span^®^ 80	3.5
Plantasens^®^ HE20	3.5
Watermelon extract	1
Water	q.s. 100

### 2.8 Determination entrapment efficiency of watermelon extract‐loaded NLCs

The entrapment efficiency was evaluated by measuring the amount of free lycopene in NLC dispersion according to Liu and Wu method with some modifications (Liu & Wu, 2010). The WH‐loaded NLCs (1 ml) were carefully weighed and mixed with 4 ml of hexane as extracting solvent, then vortexed for 1 min, and remained intact in hexane for 30 min. The sample was centrifuged at 3,000 rpm/min for 3 min, and then, supernatant was separated and detected by HPLC for free lycopene [W_free_]. Second part, amount of total lycopene in this formulation was determined by dispersed the WH‐loaded NLCs (1 ml) in hexane and ethanol with ratio of 6:4. The mixture was sonicated for 20 min and centrifuged at 7,000 rpm/min, 25°C for 30 min. The supernatant was kept and analyzed by HPLC as total lycopene [W_total_]. Finally, the percentage of entrapment efficacy was calculated according to the following equation:

(4) $$\text{Entrapment}\;\;\text{efficiency}\;\left(\%\text{EE}\right)=\left(W_{\text{total}}‐W_{\text{free}}\right)/W_{\text{total}}\times \text{1}00$$

where W_total_ is the amount of total lycopene in formulation and W_free_ is the amount of free lycopene in the formulation.

### 2.9 Stability test of watermelon extract‐loaded NLCs

The WH‐loaded NLCs were tested stability by keeping in amber glass bottle and stored under various conditions including heating–cooling cycling for 6 cycles, room temperature (RT or 30 ± 0.5°C), 4 ± 0.5°C and 45 ± 0.5°C for 3 months. Physical appearance, particle size, PDI, zeta potential, and lycopene content before and after stability test were analyzed.

For lycopene content, the amount of lycopene in formulation was analyzed by HPLC. After the stability test at each condition, the 1 ml of WH‐NLCs was dissolved with 4 ml of hexane: ethanol (6:4) and sonicated for 20 min. The supernatant was obtained and analyzed lycopene content by HPLC. The results were shown as the percentage remaining of lycopene content in the WH‐NLCs compared with the WH extract. The percentage remaining of lycopene content in each formulation was calculated from the following equation:

(5) $$\%\text{Remaining}=\frac{\text{The}\;\;\text{amount}\;\;\text{of}\;\;\text{lycopene}\;\;\text{in}\;\;\text{formulation}\;\;\text{after}\;\;\text{stability}\;\;\text{test}\times \text{1}00}{\text{The}\;\;\text{amount}\;\;\text{of}\;\;\text{lycopene}\;\;\text{in}\;\;\text{formulation}\;\;\text{at}\;\;\text{initial}}$$

### 2.10 Morphology of watermelon extract‐loaded NLCs

The morphology of WH‐NLCs was determined using transmission electron microscopy (TEM) (JEM‐2010 electron microscope, Jeol Ltd., Japan). The diluted formulation was dropped on a copper grid. After 60 s, the excess sample was removed with filter paper. The grid was rinsed with deionized water to remove any impurities and then was blotted with filter paper. As a negative staining agent, 2% phosphotungstic acid solution was added on the grid for 60 s. The excess stain was removed by touching the edge of filter paper. The grid was dried at room temperature overnight. The images of the formulation were captured using TEM at 80 kV at 20000X magnification (Singh et al., 2017).

### 2.11 In vitro release study of watermelon extract‐loaded NLCs

The release profile of lycopene from the WH extract in solution and the WH‐loaded NLCs was performed according to previous study with some modification (Okonogi & Riangjanapatee, 2015). The WH extract solution and the WH‐loaded NLCs were poured inside pre‐activated dialysis bag (molecular weight cut off 12,000 Da). Then, it was placed in propylene glycol that used as dissolution medium. The acceptor chamber was maintained at 37 ± 0.5°C and stirred with appropriate speed. The sampling (1 ml) was collected at 30 min, 1, 2, 4, 6, 8, 24, and 48 hr and replenished by fresh propylene glycol at definite time intervals. Sample was filtered with syringe filter 0.22 µm and analyzed lycopene content by HPLC. Lycopene content release from each sample was obtained and compared with each other.

### 2.12 Statistical analysis

All experiments in this study were done in triplicate. Statistical analysis was utilized through using IBM SPSS statistics 25 software. It was carried out using one‐way analysis of variance (ANOVA) to evaluate statistically significant difference followed by multiple comparisons (Tukey's test) between groups. Paired sample *t* test was used to evaluate significant difference between before and after stability test at *p* value less than 0.05.

## 3 RESULTS AND DISCUSSION

### 3.1 Analysis of lycopene content of watermelon extract

**FIGURE 1:**
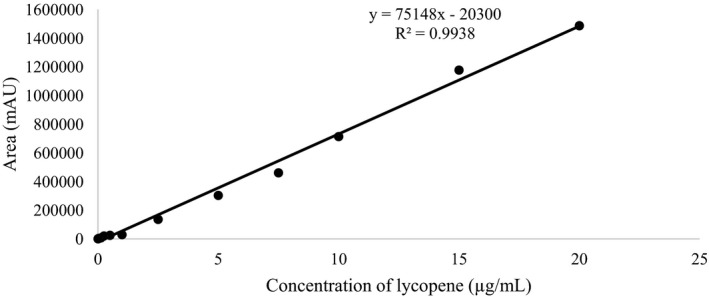
Lycopene standard calibration curve

**FIGURE 2:**
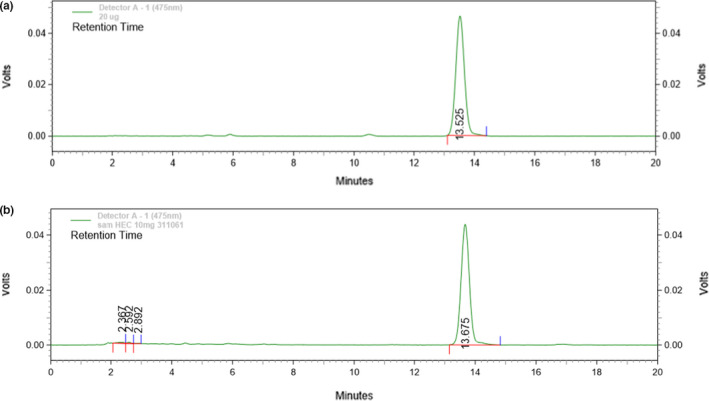
HPLC chromatograms of (a) lycopene and (b) WH extract

Freeze‐dried watermelon was extracted by Soxhlet's extraction using tri‐mixture solvents (hexane: acetone: ethanol at ratio of 2:1:1). The percentage yield of watermelon extract (WH) was 4.43 ± 1.02 and physical characteristic was sticky liquid with yellow to red color. HPLC analysis of the watermelon extract was performed and compared with lycopene standard (Figure 1). The retention time of lycopene standard and the WH extract was found at 13.525 min and 13.675 min (Figure 2). The WH extract contained 1.197 ± 0.19 µg lycopene to 1 mg extract. Previous reports suggested that lycopene is a strong antioxidant that showed higher free radical scavenging activity than β‐carotene and tocopherol (Kyriacou et al., 2018; Naz et al., 2014). The abundance of lycopene in watermelon extract is an interesting raw material of functional food and cosmetic. The previous studies had analyzed the antioxidant ability of watermelon extracts. The results indicated that the average antioxidant activity determined using cupric reducing antioxidant capacity assay ranged between 40.13 to 84.05 µmol Trolox equivalents (TE)/100 g fresh weight (Choudhary et al., 2015). The highest percentage DPPH radical scavenging activity in mesocarp was 96.76±0.61% at 125 µg/ml (Asogwa et al., 2020).

### 3.2 Preparation of unloaded NLCs

**FIGURE 3:**
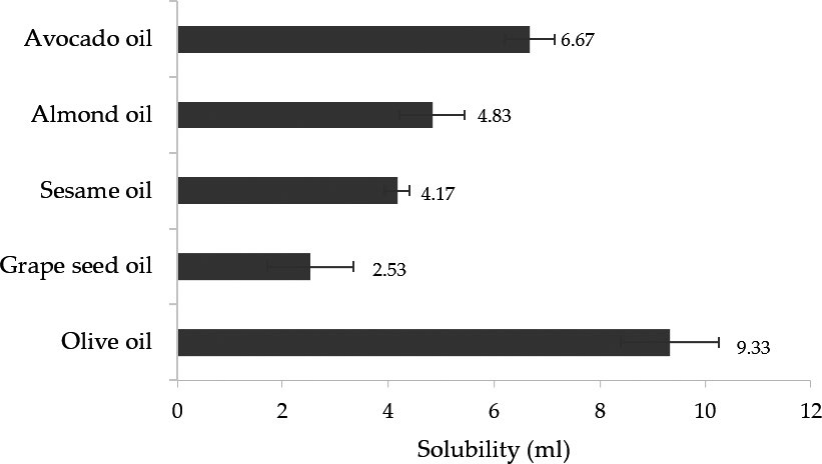
Solubility of the WH extract in liquid lipids (*n* = 3)

Cocoa butter was used as solid lipid and liquid lipids were selected from solubility and antioxidant activity. The results are shown in Figure 3 and Table 3. The WH extract could dissolve in liquid lipids following this order: grape seed oil >sesame oil >almond oil >avocado oil >olive oil. All liquid lipids were tested antioxidant activity by DPPH assay and lipid peroxidation inhibition assay. From both assays, sesame oil and grape seed oil showed the highest antioxidant activity with no significant difference at *p* > .05. The result related to previous study that grape seed oil showed the strongest antioxidant activity when compared with other fruits (Songsermsakul et al., 2013). Therefore, grape seed oil was selected to use as liquid lipid for development of NLCs due to solubility property and antioxidant ability to protect active component.

**TABLE 3:** Antioxidant activity of liquid lipids evaluated by DPPH assay and lipid peroxidation inhibitory assay (*n* = 3)

Liquid lipid	DPPH assay (% inhibition)	Lipid peroxidation inhibitory assay (% inhibition)
Olive oil	3.29 ± 2.50^a^	25.88 ± 2.52^a^
Grape seed oil	21.77 ± 1.21^c^	47.78 ± 5.53^c^
Sesame oil	19.05 ± 3.01^c^	53.27 ± 3.97^c^
Almond oil	10.69 ± 1.22^b^	37.06 ± 5.86^b^
Avocado oil	4.25 ± 1.75^a^	34.68 ± 7.15^b^

Note

Values are mean ± S.D. from triplicate. Different letters in the same column indicated significant differences (*p* < .05) in each protocol.

### 3.3 Development and characterization of unloaded NLCs

Unloaded NLCs composed of solid lipid, liquid lipid, and emulsifier as main compound. Cocoa butter and grape seed oil were used as solid lipid and liquid lipid. After obtained pre‐emulsion, it was reduced particle size by higher pressure homogenizer. In this experiment, condition of high pressure homogenizer was fixed with pressure 500 bar 8 cycles. During the homogenization process, particles break at imperfections of their crystal structure. The final particle size could be affected by the influence of process parameters of high pressure homogenizer including pressure and cycles. Amount of appropriate total lipid was chosen for loading the WH extract in this study following lipophilic property of lycopene in extract (Sirikhet et al., 2019). In previous experiment, the unloaded NLCs with solid lipid: liquid lipid ratio of 3:1 showed the smallest size and narrow PDI value (data not shown). In addition, this ratio had ability to generate nonperfect lattice and forming an amorphous structure, which brought more space for entrap active compound. Therefore, this ratio was chosen to study effect of emulsifier to produce unloaded NLCs.

Plantasens^®^ HE20 and Span^®^ 80 were used in this formulation in ratio of 1:1. Increasing of emulsifier concentration from 2.5% to 7% was represented as formulation A1 (2.5%), A2 (5%), and A3 (7%). The results are shown in Table 4. The formulation A3 generated the smallest particle size (122.40 ± 1.44 nm) due to the decreasing of interfacial tension and breaking down lipid droplets to a smaller size by emulsifier. Incorporated emulsifier in NLCs can break the repulsive force among nanoparticles and reduce the interfacial tension as well as the surface energy of NLCs (McClements, 2011). Therefore, the concentration of emulsifier is one of a critical factor that affects to stability and optimal physicochemical properties of NLCs (Hosny et al., 2015). In addition, a suitable amount of emulsifier can prevent coalescence of NLCs. For zeta potential value of the NLC formulations, they were highly negatively charged in range of −34.43 ± 0.25 to −45.40 ± 1.82 mV, which indicated good stability due to charge repulsion. Zeta potential is electrostatic charge on the surface of particles which can forecast the stability of NLCs. The zeta potential value should lie between the ranges of ≥+30 mV to ≥‐30 mV for good stability. High zeta potential can prevent aggregation and flocculation of particles and also stabilize the nanoparticle dispersion (How et al., 2011). From the results that mentioned above, the formulation A3 was chosen for loading the WH extract due to the smallest particle size, narrow PDI and high zeta potential.

**TABLE 4:** Particle size, PDI, and zeta potential of unloaded NLCs with different concentration of emulsifier (*n* = 3)

Unloaded NLCs	% emulsifier	Particle size (nm)	PDI	Zeta potential (mV)
A1	2.5	155.97 ± 0.15^c^	0.215 ± 0.009^b^	−34.43 ± 0.25^a^
A2	5	130.10 ± 2.86^b^	0.137 ± 0.035^a^	−44.13 ± 1.97^b^
A3	7	122.40 ± 1.44^a^	0.138 ± 0.012^a^	−45.40 ± 1.82^b^

Note

Values are mean ± S.D. from triplicate. Different letters in the same column indicate significant difference (*p* < .05)

### 3.4 Preparation and characterization of the WH‐loaded NLCs

The WH extract (1% w/w) was dissolved in 20% Tween^®^ 20 and lipid phase of formulation A3. Before the NLC preparation, lycopene content in extract was 1.2 mg. After the NLC preparation, the WH‐loaded NLCs were characterized particle size, PDI, and zeta potential by Zetasizer and analyzed lycopene content by HPLC. The results showed that particle size was 130.17 ± 0.72 nm, PDI value was 0.11 ± 0.032, and zeta potential was −44.00 ± 2.55 mV. The WH‐loaded NLCs showed acceptable nanosize range, narrow PDI, and high zeta potential. Lycopene content after the NLC preparation was 1.1 mg which degraded during formulation process approximately 8%.

### 3.5 Entrapment efficiency of the WH‐loaded NLCs

Percentage of entrapment efficiency (%EE) was tested from the above depicted methodology. The mixture of hexane and ethanol could effectively extract lycopene and dissolve lipid matrix of NLCs for analyzing the amount of the total lycopene in formulation. The % EE of WH‐loaded NLCs was 90.26 ± 1.10. The WH‐loaded NLCs indicated high entrapment efficient because it composed of high amount of solid lipid and appropriate liquid lipid. Incorporation of liquid lipid with more solid lipid could generate high disturbance in the crystal structure therefore the resultant matrix of the lipid particles demonstrates more imperfect crystal lattice and providing enough space for the active compound molecule to accommodate (Souto et al., 2004). Moreover, entrapment efficacy depends on the amount of the total lipid and the solubility of the compound in lipid. More concentration of active compound that could be soluble in the lipid components generated high active compound entrapment (Singh et al., 2016).

### 3.6 Stability study

**FIGURE 4:**
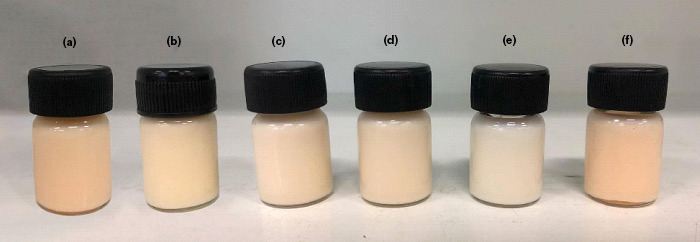
The WH‐loaded NLCs before and after storage at various conditions. (a): before, (b): heating–cooling condition (6 cycles), (c): 30°C with light, (d): 30°C with dark, (e): 45°C, and (f): 4°C for 3 months

**FIGURE 5:**
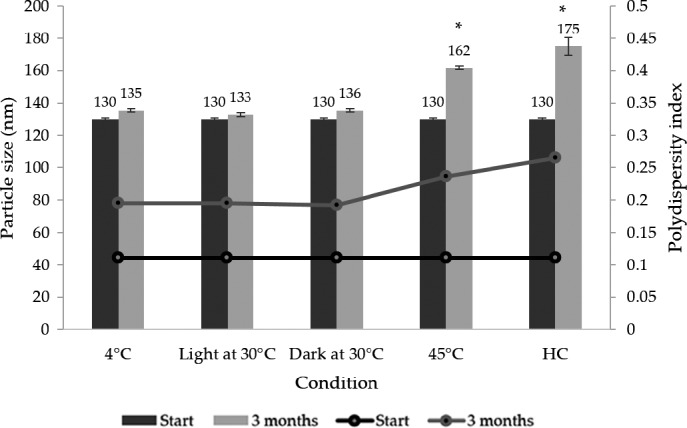
Particle size and PDI of the WH‐loaded NLCs before and after stability test (*n* = 3). Asterisk (*) indicates significant difference (*p* < .05) when compared between before and after stability test

The WH extract and the WH‐loaded NLCs were kept at various conditions, heating–cooling (HC), 4 ± 0.5°C, 30 ± 0.5°C with light and dark conditions, and 45 ± 0.5°C for 3 months. Physical appearance, particle size, PDI, zeta potential, and lycopene content before and after stability test were analyzed. The physical appearance of WH‐loaded NLCs including pH value did not change after storage for 3 months. However, color changed with unappropriated condition as shown in Figure 4. The particle size, zeta potential, and PDI were 130.17 ± 0.72 nm, −44.00 ± 2.55 mV, and 0.110 ± 0.032, respectively at initial. After stability test, the particle size significantly increased (*p* < .05) after heating–cooling and 45°C, whereas it did not change after storage at 4°C and room temperature (Figure 5). It could be indicated that the WH‐loaded NLCs slightly changed physical appearance at high temperature for the long storing period but no coalescence or phase separation occurred.

**FIGURE 6:**
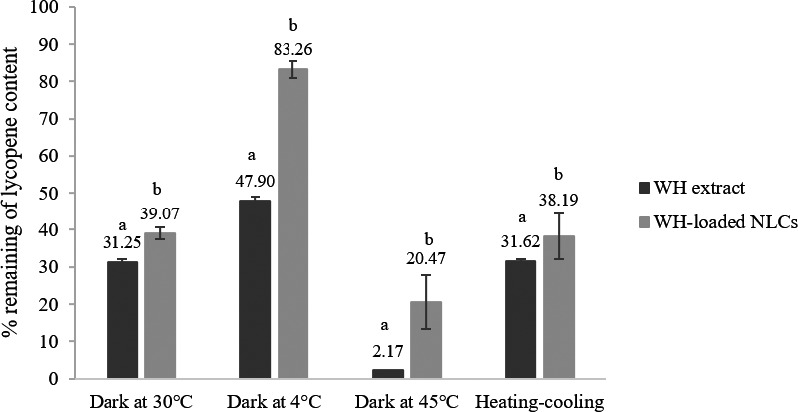
Percentage remaining of lycopene content in WH extract and WH‐loaded NLCs before and after stability test (3 months) at various conditions (*n* = 3). a and b indicate significant difference (*p* < .05) in each condition

**FIGURE 7:**
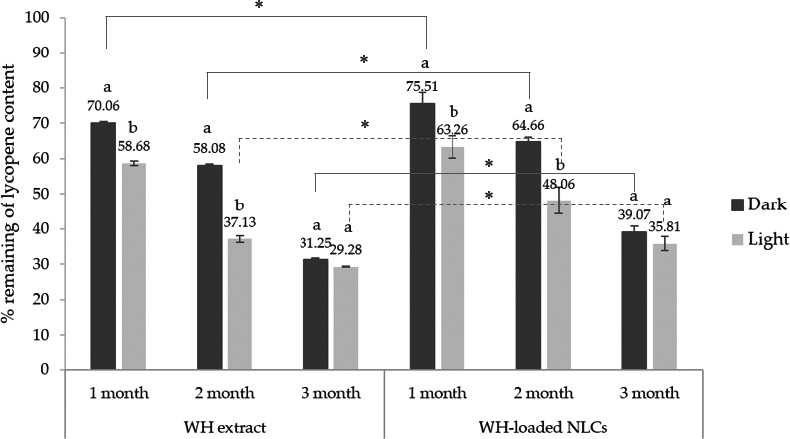
Percentage remaining of lycopene content in WH extract and WH‐loaded NLCs after storage for 3 months compared between dark and light condition at room temperature (*n* = 3). a and b indicate significant difference (*p* < .05) in each month. Asterisk (*) indicates significant difference (*p* < .05) when compared between WH extract and WH‐loaded NLCs at the same condition

The lycopene contents of WH extract and WH‐loaded NLCs were compared by HPLC. At initial (start), the lycopene content was quoted as 100%. The results were presented in percentage remaining of lycopene content as shown in Figures 6 and 7. The lycopene contents in WH extract were dramatically decreased when kept in all conditions for a long storage of time. The highest percentage remaining of lycopene content in WH extract was found at 4°C (47.90 ± 0.98%) followed by room temperature (31.25 ± 0.95%) and HC condition (31.62 ± 0.43%). In contrast, the WH‐loaded NLCs kept in similar condition presented significantly higher percentage remaining of lycopene content than the WH extract at all conditions (*p* < .05). Especially at 4°C, lycopene content of the WH‐loaded NLCs was 83.26 ± 2.30% that exhibited the highest percentage remaining of lycopene content. Percentage remaining of lycopene content in WH extract and WH‐loaded NLCs were significantly decreased after storage at 45°C (*p* < .05) that exhibited the lowest percentage remaining 2.17 ± 0.03 and 20.47 ± 7.23%, respectively. From the results, it was confirmed that the NLCs could improve the stability and protect sensitive bioactive compound from temperature and unpleasant conditions better than crude extract. However, the WH‐loaded NLCs should be kept at 4°C to remain the highest lycopene content. Concordantly with previous study showed that lycopene loaded chitosan alginate nanoparticles exhibited greater stability after storage for 12 weeks in refrigerator than storage in dark and visible light at room temperature (Limvongsuwan, 2005).

For effect of light, the WH extract and the WH‐loaded NLCs gave similarly tendency results. Lycopene content was significantly decreased (*p* < .05) when kept in light condition compared with dark condition after storage at 1–2 months as shown in Figure 7. However, lycopene content of all samples was not significantly different between light and dark condition when kept for 3 months. So light was rapidly affected lycopene stability in initial storage but not affect for long time storage. In addition, the percentage remaining of lycopene content in the WH‐loaded NLCs was degraded less than lycopene in the WH extract due to the light protection of solid lipid of NLC formulation. It can be concluded that light and temperature more than 30°C with long‐term storage affected on degradation of lycopene. Heat and light can induce oxidation and isomerization of lycopene from all‐trans‐form to cis‐form. So, a shorter period of time of heating and less light irradiation in processing and storage can reduce lycopene degradation to a great extent (Shi et al., 2003).

### 3.7 Morphology of the unloaded NLCs and the WH‐loaded NLCs

**FIGURE 8:**
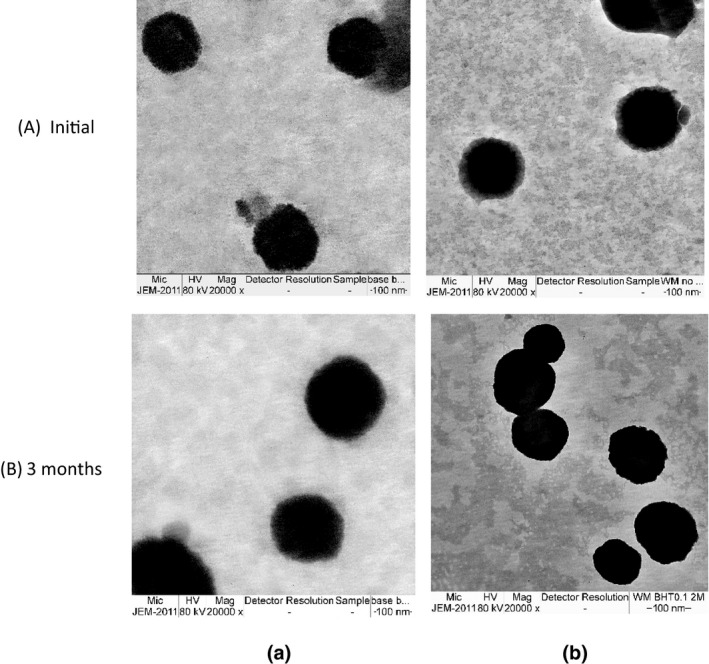
The TEM images of unloaded NLCs (a) and WH‐loaded NLCs (b) at initial (A) and after 3 months of storage (B)

TEM analysis revealed the morphology of nanoparticles. The TEM images of unloaded NLCs (formulation A3) and WH‐loaded NLCs are illustrated in Figure 8. They showed a spherical shape with 100–150 nm in diameter. The results were consonance with the particle size measurement by Zetasizer^®^. The morphology of formulation A3 and WH‐loaded NLCs after storage for 3 months was not changed when compared with initial. The TEM observations were confirmed the uniformly well‐formed spherical shapes of NLCs in both formulations.

### 3.8 In vitro release study of the WH‐loaded NLCs

**FIGURE 9:**
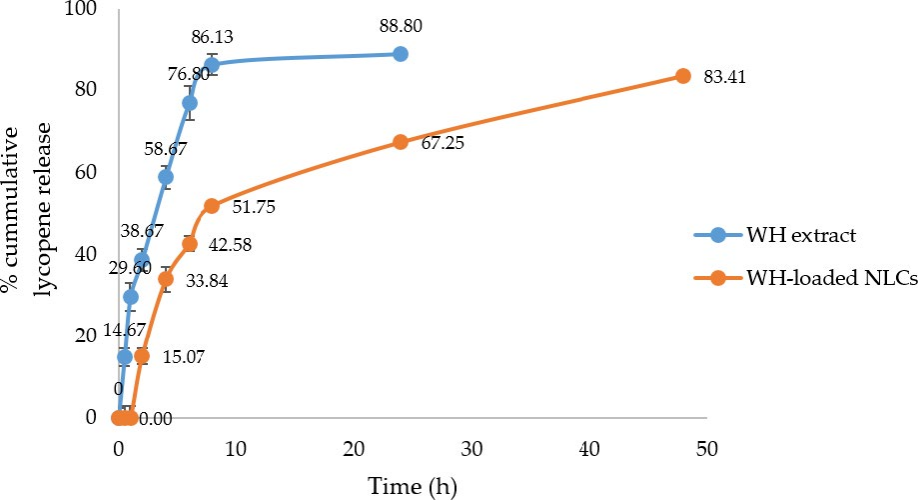
In vitro release profile of the WH extract in solution and the WH‐loaded NLCs (*n* = 3)

The lycopene release profiles from WH extract in solution and the WH‐loaded NLCs were evaluated by a dialysis bag method. Lycopene is very low water soluble so its tendency to remain in lipid nanoparticles. Propylene glycol was used as release media for solving this problem due to penetration enhancer property. Analysis of lycopene release profile was performed over 24 hr by HPLC.The amount of lycopene release was compared to the initial dose. The percentage of lycopene release from WH extract in solution and WH‐loaded NLCs is shown in Figure 9. The WH extract in solution exhibited a rapid release within 8 hr and showed percentage of cumulative lycopene release at 88.80 ± 2.56 after 24 hr, whereas lycopene release from the NLC formulation showed a continuously release pattern at the initial stage and followed by a prolonged release until 48 hr at 83.41 ± 1.92%. The results confirmed that the WH‐loaded NLCs can generate prolonged release of lycopene. The control release pattern is occurred because more concentration of lycopene is dissolved in the lipid core so it continually slow release though the cellulose pores (Müller et al., 2006).

## 4 CONCLUSIONS

The WH‐loaded NLCs were successfully prepared to enhance the stability and prolonged release profile of lycopene. The WH‐loaded NLCs consisted of cocoa butter and grape seed oil as solid lipid and liquid lipid in ratio of 3:1 (10%w/w of total lipid). Span^®^ 80 and Plantasens^®^ HE20 at 7%w/w were the best concentration to prepare appropriated NLCs. The WH‐loaded NLCs showed small particle size, narrow PDI, value and high zeta potential. It revealed a spherical morphology and achieved high‐lycopene entrapment efficiency. In addition, it also showed good property to protect unstable of lycopene after storage especially at 4°C for 3 months. Therefore, this NLC formulation is a promising delivery system with safety ingredient and easy process for improve stability of WH extract and other unstable extracts. Furthermore, it can further study for use in industry or high scale production.

## CONFLICTS OF INTEREST

The authors declare that there is no conflict of interest regarding the publication of this paper.
