# ERRATUM

**DOI:** 10.1002/fsn3.2555

**Published:** 2021-09-15

In the article by Kanokwan Kiattisin et al., (2021) there is a change in the affiliation of the corresponding author. The affiliation should be read as:

2. Innovation Center for Holistic Health, Nutraceuticals, and Cosmeceuticals, Faculty of Pharmacy, Chiang Mai University, Chiang Mai, Thailand
